# Laboratory diagnosis of tuberculous meningitis in human immunodeficiency virus–seropositive patients: Correlation with the uniform case definition

**DOI:** 10.4102/sajid.v35i1.135

**Published:** 2020-08-26

**Authors:** Mohammed Mitha, Melendhran Pillay, Julie Y. Moodley, Yusentha Balakrishna, Nathlee Abbai, Smita Bhagwan, Zaynah Dangor, Ahmed I. Bhigjee

**Affiliations:** 1 Department of Pulmonology, Inkosi Albert Luthuli Central Hospital, Durban, South Africa; 2 National Health Laboratory Services Department, University of KwaZulu-Natal, Durban, South Africa; 3 Medical Microbiology Department, University of KwaZulu-Natal, Durban, South Africa; 4 Biostatistics Unit, South African Medical Research Council, Durban, South Africa; 5 School of Clinical Medicine Research Laboratory, Nelson Mandela School of Medicine, University of KwaZulu-Natal, Durban, South Africa; 6 Department of Neurology, Nelson R Mandela School of Medicine, University of KwaZulu-Natal, Durban, South Africa

**Keywords:** HIV, tuberculous meningitis, uniform case definition, laboratory diagnosis, World Health Organization

## Abstract

**Background:**

Laboratory confirmation of the diagnosis of tuberculous meningitis (TBM) has always been problematic. Using the uniform case definition suggested by Marais et al., we determined the sensitivity of a variety of laboratory tests.

**Methods:**

Human immunodeficiency virus (HIV)–seropositive patients suspected of having subacute meningitis were included in the study. Using the uniform case definition, patients were divided into possible and probable cases of TBM. The following specific tests were done on the cerebrospinal fluid (CSF): layered Ziehl–Neelsen (ZN) staining, CSF culture and a panel of nucleic acid amplification tests (NAAT) consisting of the GenoType MTBDRplus assay, Cepheid Xpert MTB/RIF, the MTB Q-PCR Alert (Q-PCR) and the loop-mediated isothermal amplification (LAMP) assay. The sensitivity of each test was compared to the case definition and to each other.

**Results:**

A total of 68 patients were evaluated. Using the uniform case definition only, without any of the specific laboratory tests, there were 15 probable cases (scores > 12) and 53 possible cases (scores 6–11) of TBM. When the uniform case definition was tested against any laboratory test, 12 of the 15 (80%) probable cases and 26 of the 53 (49.1%) possible cases had laboratory confirmation. When each test was compared to any other test, the sensitivities for the Xpert MTB/RIF, GenoType MTBDRplus, CSF culture, Q-PCR, LAMP and ZN layering were 63.2 (46.0–78.2), 76.3 (59.8–88.6), 65.7 (47.8–80.9), 81.1 (64.8–92.0), 70.3 (53.0–84.1) and 55.6 (38.1–72.1), respectively.

**Conclusion:**

In this study, the GenoType MTBDRplus and the Q-PCR tests performed better than the Xpert MTB/RIF. Because the Xpert MTB/RIF is not good enough to ‘rule out’ TBM, a negative result should be followed up by another NAAT, such as the GenoType MTBDRplus or Q-PCR. The LAMP assay may be considered as the first test in resource-poor settings. At the time of the study, we did not have access to the Xpert MTB/RIF Ultra, which has now been recommended by the World Health Organization as the test of first choice. However, even this test has a similar limitation as the Xpert MTB/RIF, with two recent studies showing variable results.

## Introduction

In 2018, an estimated 10 million people fell ill with tuberculosis (TB), the majority (90%) of whom were adults.^1^ Where the human immunodeficiency virus (HIV) prevalence is > 1%, the risk of active tuberculosis is 20.6%.^1^ Extra-pulmonary TB (EPTB) constitutes about 14% – 24% of all reported cases of TB in high-prevalence areas.^1^ The occurrence of EPTB is higher in HIV-positive patients because of more frequent and earlier dissemination of the organism.^2^ Tuberculous meningitis is the most devastating form of TB infection because of the high morbidity and mortality. Human immunodeficiency virus–co-infected TBM patients are more vulnerable, with an estimated mortality of more than 60%.^3^

The primary reasons for this high morbidity and mortality are the delay in suspecting the diagnosis, the difficulty confirming the diagnosis once suspected and subsequent delay in initiating therapy. The initial presentation, which may last for at least a week, is non-specific, comprising malaise, fever, weight loss and gradual onset of headache. These features could all be mistaken for a mild viral infection. The various conventional TB laboratory tests, though specific, lack sensitivity. The current ‘gold standard’, which is TB culture, takes too long to be useful in deciding on initiation of therapy.

Various attempts have been made to improve the early diagnosis of TBM using clinical or laboratory criteria or both. However, in the individual HIV-infected adult patient, atypical presentation of the routine clinical and basic laboratory features occur too often for these features to be really useful. Using extra-neurological evidence of active TB disease as a criterion for the diagnosis of probable TBM is also problematic. Bhagwan and Naidoo found that the most common cause of subacute meningitis in HIV-infected patients who had sputum-positive pulmonary TB was cryptococcal meningitis.^4^ Further, a significant proportion of patients have more than one infection at any one site (personal observation).

Attempts to improve laboratory diagnosis include variations in techniques for smear, culture and nucleic acid amplification tests (NAAT) and immunological and biochemical assays. Individually, these laboratory tests have sensitivities around 58% and specificities of 94% (summarised in Marais et al.^5^). The sensitivity improves to 82% and specificity to 100% if concurrent tests are used to exclude acute bacterial (Gram stain) and cryptococcal infections (antigen detection). In summary, the major drawback of all the clinical algorithms, the non-specific laboratory tests as well as the specific tests, is that none of them reliably ‘rules out’ a diagnosis of TBM.

In 2010, Marais et al. suggested a uniform case definition of TBM for use in clinical research.^5^ They categorised patients into definite (demonstration of the acid fast bacilli either by smear, culture or NAAT), probable (score > 10/12) and possible (score 6–9/6–11) TBM cases.

Together with the uniform case definition, we undertook a prospective study to investigate the diagnostic performance (sensitivity) of the different laboratory tests used to diagnose TBM. The techniques or assays tested were cerebrospinal fluid (CSF) layering, CSF culture, the GenoType MTBDRplus assay, the Cepheid Xpert MTB/RIF system, the MTB Q-PCR Alert (Q-PCR) and the loop-mediated isothermal amplification (LAMP) assay.

## Patients, materials and methods

The study was undertaken in the Neurology Department at Inkosi Albert Luthuli Central Hospital (IALCH) in Durban, South Africa, which together with the Neurology Department of Grey’s Hospital, situated 90 km away, provides neurological services to 11.5 million individuals in the province of KwaZulu-Natal.^6^

We included patients who were suspected of having subacute meningitis. They were referred from peripheral hospitals to IALCH if they had symptoms of meningitis and one or more of the following: meningism, disturbance of consciousness, focal signs or seizures. Patients were excluded from the study if there was any contraindication for a lumbar puncture or the patients were on anti-TB treatment for more than 1 week. Consent was obtained from the patient or next of kin.

All patients were recruited prospectively and consecutively from 2010 to 2015. A clinical assessment was conducted with special reference to the uniform case definition of Marais et al.^5^ All patients underwent either a computer tomographic (CT) scan or magnetic resonance imaging (MRI) scan of the brain. The imaging criteria that were recorded were hydrocephalus, basal meningeal enhancement, tuberculoma and infarct. A chest radiograph was reported as being normal or abnormal. If the chest radiograph was abnormal, it was recorded as either showing active disease, old disease or miliary disease.

Blood samples were taken for the following tests: full blood count, urea and electrolytes, erythrocyte sedimentation rate, glucose, syphilis serology, HIV serology (special additional consent obtained), HIV viral load and CD4 cell count. A routine CSF sample was subjected to biochemistry (glucose and protein), microscopy (polymorphs, lymphocytes and red blood cells) Gram and auramine stains, bacterial and mycobacterial liquid cultures using the BACTEC MGIT 960 system and drug susceptibility testing using Middlebrook 7H10 agar, the cryptococcal antigen test, the fluorescent *Treponema pallidum* test and polymerase reaction (PCR) assays for herpes simplex virus, varicella zoster virus and cytomegalovirus. An additional 10 ml of CSF was collected for the Ziehl–Neelsen (ZN) layering smear technique, the GenoType MTBDRplus PCR assay, the Cepheid Xpert MTB/RIF system, Q-PCR and the LAMP test. The samples for the NAAT were frozen at –20°C and analysed at different times in batches.

### Cerebrospinal fluid layering method

Cerebrospinal fluid was aliquoted into 50-ml polypropylene tubes and centrifuged at 3000 g for 15 min. A drop of CSF was placed on the prepared slide and then dried on a hot plate at 65°C – 75°C. This was repeated four to five times depending on the sample size. The slides were then stained using ZN stain. Analysis of the results took approximately 20 min, to read and interpret each slide.

### Sample preparation for deoxyribonucleic acid studies

Up to 3 ml of CSF was spun at 3000 g for 15 min. The supernatant was decanted and the pellet resuspended in 2 ml of reagent buffer. Given the paucibacillary nature of the specimen, centrifugation was able to provide a more concentrated yield. Buffers such as phosphate-buffered saline (PBS) (0.5 ml) were added to maintain cell integrity prior to automated deoxyribonucleic acid (DNA) extraction within the Xpert MTB/RIF system. The Xpert MTB/RIF protocol also required a final volume of 2 ml to be transferred to the cartridge for final extraction, amplification and *Mycobacterium tuberculosis* (MTB) detection.

### The GenoType MTBDRplus assay

Deoxyribonucleic acid extraction and the assay were conducted as per the Hain Lifescience methodology (Nehren, Germany). *Mycobacterium tuberculosis* DNA was amplified using specific optimised primers as per the GenoType MTBDRplus assay (Hain Lifescience). Amplification of the 23S ribosomal ribonucleic acid sequence as well as the *rpoB, katG* and *inhA* genes encoding the rifampicin and isoniazid drug target areas was performed simultaneously. A negative control containing no DNA template was included with each run. Deoxyribonucleic acid hybridisation patterns were visualised chromogenically to determine the species of interest, and sensitivities to rifampicin and isoniazid were determined through the absence or presence of wild-type and mutant probes, respectively.

### Cepheid Xpert MTB/RIF system

This test is a fully automated NAAT that detects the presence of the *rpoB* gene of *Mycobacterium tuberculosis* complex (MTBC) and associated mutations that confer rifampicin resistance.

The GX 2.1 software package was used to analyse the samples within the cartridges for the presence of MTB. Processing was initiated by scanning the cartridge barcode into the system. The cartridge was placed in a module for automatic processing.

The final results were determined from the measured fluorescent signals of amplified MTB probes.

### MTB Q-PCR Alert

The MTB Q-PCR Alert (Nanogen Advanced Diagnostics, Trezzano sul Naviglio, Italy) was performed on the same DNA elutes as used for the GenoType MTBDRplus assay. Detection of MTB was determined by a positive amplification of insertion sequence 6110 (IS*6110*) using the specific primers and probes provided; 1 µl of internal control and 4 of µl DNA template were added to each PCR reaction mix (25 µl). The Rotor-Gene Q MDx instrument was used for real-time thermal cycling, amplification and detection of IS*6110*.

### Loop-mediated isothermal amplification

A 600-µl sample of CSF was centrifuged at 4300 g for 20 min at room temperature. Following centrifugation, the supernatant was decanted, and 60 µl of PBS was added to the sample pellet. The pellet was resuspended in the buffer by briefly vortexing it. The homogenised samples were then used directly for the LAMP assay.

This test is a manual NAAT that detects the MTBC genome but does not identify drug resistance. The assay was performed according to the manufacturer’s instructions, and results were recorded as positive or negative based on fluorescence under UV light.

The laboratory personnel were blinded to the clinical details. The different laboratory staff worked independently from each other.

The data were analysed using Stata 15 (StataCorp, College Station, TX, USA). Whereas CSF TB culture positivity would be the gold standard, it has low sensitivity. Therefore, the uniform case definition was used in a slightly different format. Using the uniform case definition but excluding the test under question, the cases were divided into probable and possible cases. This approach also allowed for the evaluation of the definitions of probable and possible cases.

The frequency and percentage of positive results found by each test were described. The sensitivities of each test were determined, using any positive test as the gold standard.

### Ethical consideration

The study was approved by the Biomedical Research Ethics Committee of the University of KwaZulu-Natal (BF016/010 & BE235/16).

## Results

Only those patients who were negative for other causes of subacute meningitis such as syphilis and cryptococcal meningitis (see above for blood and CSF tests done – results not shown) were subjected to further analysis. The number of patients with other diagnoses was not recorded.

A total of 68 patients were evaluated. Using the uniform case definition^5^ only, without any of the specific laboratory tests, there were 15 probable cases (scores > 12) and 53 possible cases (scores 6–11) of TBM. A further three had scores of 3, 4 and 5. The specific laboratory tests of these three cases were all negative. As the diagnoses in these cases were uncertain, they were excluded from further analysis.

Table 1 shows the frequency of positive results for each test. When the uniform case definition was tested against any laboratory test, 12 of the 15 (80%) probable cases and 26 of the 53 (49.1%) possible cases had laboratory confirmation. Table 2 gives the results obtained in determining definite cases after excluding the test of interest. In this instance each test was assessed against the remaining five tests. The question asked was the following: If any of the remaining five tests was positive, did the test of interest also return a positive result? The Q-PCR test performed best after the GenoType MTBDRplus test.

**TABLE 1:** Frequency of positives in the probable and possible groups using the uniform case definition.

Test	Possible (*n* = 53)		Probable (*n* = 15)		Total	
	*n*	%	*n*	%	*n*	%
Xpert MTB/RIF†	16	30.2	8	53.3	24	35.3
GenoType MTBDRplus-MTB†	19	35.9	10	66.7	29	42.7
TB culture	18	34.0	8	53.3	26	38.2
Q-PCR MTB†	21	39.6	10	66.7	31	45.6
LAMP-MTB†	17	32.1	10	66.7	27	39.7
ZN layering technique	14	26.4	8	53.3	22	32.4
Any of the six tests positive†	26	49.1	12	80.0	38	55.9

LAMP-MTB, loop-mediated isothermal amplification-*Mycobacterium tuberculosis*; MTB/RIF, *Mycobacterium tuberculosis*/rifampicin; PCR-MTB, polymerase reaction-*Mycobacterium tuberculosis*; Q-PCR MTB, Q-polymerase reaction *Mycobacterium tuberculosis*; TB, tuberculosis; ZN, Ziehl–Neelsen.

† , Based on 68 participants. One participant with a ‘possible’ case definition had missing data.

**TABLE 2:** Sensitivity testing: Excluding test of interest and one of the remaining five tests positive as the gold standard with prevalence (pretest odds) of tuberculous meningitis at 65% (95% confidence intervals [CIs]).

Test	Xpert MTB/RIF	GenoType MTBDRplus-MTB			TB culture		MTB Q-PCR		LAMP-MTB		ZN layering technique	

	Mean	95% CI	Mean	95% CI	Mean	95% CI	Mean	95% CI	Mean	95% CI	Mean	95% CI
**Sensitivity**	63.2	46.0–78.2	76.3	59.8–88.6	65.7	47.8–80.9	81.1	64.8–92.0	70.3	53.0–84.1	55.6	38.1–72.1

LAMP-MTB, loop-mediated isothermal amplification-*Mycobacterium tuberculosis*; MTB/RIF, *Mycobacterium tuberculosis*/rifampicin; CI, confidence interval; PCR-MTB, polymerase reaction-*Mycobacterium tuberculosis*; MTB Q-PCR, *Mycobacterium tuberculosis* Q-polymerase reaction; TB, tuberculosis; ZN, Ziehl–Neelsen.

The chest radiographs were grouped as being normal or abnormal. The abnormal ones were not further subdivided as the numbers in each subgroup were too small for analysis. Chest radiograph results were available for 56 patients. Using Fisher’s exact test, there was a significant association between chest radiographs and having any one test positive (*p* < 0.001). Most of the patients (94.7%) with an abnormal chest radiograph had at least one positive test. Also, most of the patients (56.8%) with a normal chest radiograph did not have a laboratory confirmation. Thus, an abnormal chest radiograph was likely to be associated with at least one test positive.

## Discussion

The uniform case definition has been used as the ‘gold standard’ in a few studies concerning HIV-positive patients with suspected TBM.^7,8,9^ For example Bahr et al. compared the performance of Xpert MTB/RIF against the gold standard, which resulted in a 70% sensitivity.^9^ In this study, 12 of the 15 (80%) probable cases and 26 of the 53 (49.1%) possible cases moved to the ‘definite’ category if any one of the tests was positive. The case definition is as good as any laboratory test in the probable cases but not so in the possible cases. In the HIV-positive patients, the presence of TB elsewhere (which carries a maximum category score of 4 in the uniform case definition) should be used with caution, as discussed earlier. It needs to be emphasised that the uniform case definition was designed for research purposes and should not replace clinical judgement in routine clinical practice nor require the exclusion of the number of alternate diagnoses, especially in a resource-poor setting.

The World Health Organization (WHO) endorsed the use of the Xpert MTB/RIF as the first test in the diagnosis of TBM.^10^ The Xpert MTB/RIF has a sensitivity of around 60% – 70% with a specificity of close to 100%.^7,11,12^ Use of large volumes (often not possible as CSF flow may be reduced to a mere trickle) and centrifugation may improve sensitivity.^7,11^ Whereas the Xpert MTB/RIF is an excellent ‘rule in’ test, it is a poor ‘rule out’ test. Once again, caution is advised in its use in routine clinical practice.^13^ In this study, the GenoType MTBDRplus and Q-PCR tests performed better than the Xpert MTB/RIF.

More recently, the LAMP test has been evaluated for the diagnosis of TBM. Two studies using in-house LAMP kits found sensitivities ranging from 88% to 90% compared to 52% – 80% sensitivities for PCR tests.^14,15^ We used a commercial kit (Eiken Chemical, Tokyo, Japan) and found it to be slightly superior to the Xpert MTB/RIF (see Table 1). The LAMP test is simple, quick (results within 60 min), does not require specialised equipment and is cheaper (R150.00) compared to the Xpert MTB/RIF (R187.00).^16^ Thus, LAMP has the potential to be used by any laboratory and would be a useful test in a resource-poor setting.

A second-generation Xpert MTB/RIF test, the Xpert MTB/RIF Ultra, was not available to us at the time of the study. A 2017 WHO technical report suggested 95% sensitivity for TBM.^17^ The WHO has now endorsed the Xpert MTB/RIF Ultra as the test of first choice. However, when using it as a single test for TBM in HIV-positive patients, Bahr et al.^9^ found that the Ultra had a sensitivity of 70% when tested against the uniform case definition. The sensitivity improved only by using a composite reference standard, a situation that is not practical in a busy resource-poor setting, where often there is also a lack of well-trained medical staff. Two more recent studies using different methodologies gave contrasting results with the Xpert MTB/RIF Ultra. Cresswell et al.^18^ found that the Xpert MTB/RIF Ultra was superior to the Xpert MTB/RIF, but it had a negative predictive value of 93%. However, Donavan et al.^19^ found that the Xpert MTB/RIF Ultra was not superior to the Xpert MTB/RIF when compared to the uniform case definition.

## Conclusion

This study highlights once again the difficulty in confirming a diagnosis of TBM. The different NAAT in the same sample of CSF demonstrate the considerable variation in these tests. There is an inherent danger that the inexperienced or junior clinician may rely too heavily on the Xpert MTB/RIF or the Xpert MTB/RIF Ultra to ‘rule out’ TBM. Clinical judgement is still required. We suggest that a negative Xpert MTB/RIF/Xpert or Xpert MTB/RIF Ultra be followed up by a second PCR test, either the GenoType MTBDRplus test or the Q-PCR test. In remote areas, the LAMP test may be a suitable initial test.

A limitation of this study is that we did not have a control group, which would have provided information regarding the specificity of the tests used.
